# Self-expandable stented valve with glutaraldehyde-fixed cardiac xenograft treated by novel anticalcification protocols including immunologic modification for perventricular pulmonic valve implantation

**DOI:** 10.1186/1749-8090-10-S1-A140

**Published:** 2015-12-16

**Authors:** Min-Seok Kim, Saeromi Jeong, Gi Beom Kim, Hong-Gook Lim, Yong Jin Kim

**Affiliations:** 1 Department of Thoracic and Cardiovascular Surgery, Seoul National University Hospital, 101, Daehak-ro, Jongno-gu, Seoul 110-744, Republic of Korea; 2 Department of Pediatrics, Division of Pediatric Cardiology, Seoul National University Hospital, 101, Daehak-ro, Jongno-gu, Seoul 110-744, Republic of Korea

## Background/Introduction

The conventional glutaraldehyde (GA) fixation for cardiac xenograft causes dystrophic calcification, and there is also a significant immune reaction to the galactose-α-1,3 galactose β-1,4-N-acetylglucosamine (α-Gal), leading to calcification. So, future investigation should be redirected in the quest for the αGal-free long-lasting substitutes.

## Aims/Objectives

We made the αGal-free long-lasting stented valve which is comprised of self-expanding nitinol frame containing GA-fixed xenografts treated by our novel combined anticalcification protocol. The safety and efficacy of our stented valve were evaluated using large-animal in vivo perventricular pulmonic circulatory models.

## Method

Porcine cardiac xenografts were treated by decellularization, immunologic modification with alpha-galactosidase, space filler, GA fixation in organic solvent, and detoxification. The nitinol (Nikel-Titanium, shape memory alloy) frame is comprised of a braided wire that reverts to its original shape and locks the valve into position as deployed. We manufactured the stented valve with the porcine cardiac xenografts mounted on a nitinol frame. The stented valves were implanted in ovine pulmonary valve position via perventricular approach, and durability was evaluated for 18 months after implantation.

## Results

To 18 months after perventricular pulmonic valve implantation, evaluation of echocardiography and cardiac catheterization demonstrated good hemodynamic status and function of pulmonary valve, and good endothelialization resulted in no paravalvular leakage. Durability of the porcine cardiac xenografts was well preserved without calcification.

## Discussion/Conclusion

We demonstrated the safety and efficacy of the αGal-free long-lasting stented valve with our synergistic and simultaneous employment of multiple anticalcification therapies including immunologic modification with alpha-galactosidase. The future clinical study is warranted based on these promising preclinical results using large-animal in vivo perventricular pulmonic circulatory models.

